# Alkaline–Acidic Sodium Chlorite Pretreatment of Bamboo Powder for Preparation of Excellent Mechanical, Transparent, and Biodegradable Films

**DOI:** 10.3390/polym16233361

**Published:** 2024-11-29

**Authors:** Jing Wang, Ling Sun, Ying-Ying Chu, Yong-Qi Ou, Bo-Wen Liang, Zi-Jian Shan, Chang-Bao Han, Hui Yan

**Affiliations:** 1 Key Laboratory of Advanced Functional Materials, Institute of Advanced Energy Materials and Devices, Ministry of Education, Faculty of Materials and Manufacturing, Beijing University of Technology, Beijing 100124, China; 2 Beijing Guyue New Materials Research Institute, Beijing University of Technology, Beijing 00124, China

**Keywords:** all cellulose, moso bamboo, packaging film, mechanical property, transparent

## Abstract

Bamboo is widely distributed around the world as an excellent renewable resource. However, the structural and morphological changes in the bamboo samples in extracting bamboo cellulose fiber using alkaline-acidic sodium chlorite are unclear, and the potential for preparation of cellulose packaging films is yet to be explored. In this paper, the changes in micro-morphology, chemical structure, and pyrolytic behavior of moso bamboo powder during alkaline and acidic sodium chlorite pretreatment were intensively investigated. The bamboo cellulose fiber (BC) diameter decreased from 14.41 to 11.79 µm with the treatment as a result of the removal of amorphous materials such as lignin and hemicellulose. The BC was dissolved in NaOH/urea aqueous solution, and all-cellulose composite films were obtained with excellent mechanical properties and high transparency. When the BC contents reached 4 wt%, the resulting films had a light transmittance of about 90% in the visible light range (400–780 nm), and the tensile strength was as high as 57.9 MPa, which was much higher than that of the polyethylene packaging film (PE, 35 MPa). In addition, the film also suggests superior biodegradability compared to PE films. Therefore, the current shortage of raw materials and environmental pollution faced by plastic packaging materials may be expected to gain new inspiration in this study.

## 1. Introduction

Nowadays, improving people’s living standards highlights the high demand for packaging materials for food, medical supplies, and electronic products [1,2]. At present, there are many types of packaging materials commonly used in the market, including metal, glass, paper, plastic, and other materials, of which plastic has become the most commonly used packaging material because of its low cost, excellent barrier properties, lightweight, high strength, high transparency, and other characteristics [3,4]. According to statistics, the plastics industry has grown dramatically in the past few decades. In the whole year of 2018, China’s plastic production was 60,421,500 tons, while at the same time generating waste in the range of 45–50 million tons, of which 40% are applied to the packaging industry [5]. However, plastic packaging products in the preparation process, because of the presence of additives, affect the environment as well as biologically and will have a negative impact, and petroleum-based raw materials for plastic products recycling difficulties (less than 50% of the plastic production recycling in 2018), either landfill or incineration, will produce unavoidable pollution of the environment, and incineration of dioxin on the human body has carcinogenic effects [6,7]. In summary, the threat of plastic products has become the most vital threat to solving the pollution problem on earth [8,9,10] and is also an urgent problem in the current energy crisis.

Bio-materials, especially biopolymers such as starch [11], chitosan [12], and cellulose [13], have attracted much attention due to their unique natural degradability properties. Among them, cellulose is considered the most abundant organic matter on earth. It has become a popular raw material in packaging because of its vast source and natural renewability [14,15]. Cellulose is a biomass material widely present in plant cell walls. Herbaceous plants [16], woody plants [17], and some plant products [18] can be used as a source of cellulose [19,20]. However, among the many sources, bamboo, a highly lignified herbaceous plant with a chemical composition and microstructure similar to that of wood, has a much shorter growth cycle than that of wood, generally 3–5 years [21], and is widely distributed in both tropical and subtropical regions. China possesses the largest bamboo resources in the world in terms of species and production, of which the area of moso bamboo accounts for 73% of the total bamboo forest area [22]. Therefore, there is great potential for using moso bamboo as a raw cellulose material.

The method of pretreating biomass raw materials has been deeply explored in recent years, including physical, biological, and chemical methods [23]. Although physical method pretreatment has the advantage of being environmentally friendly, it has high energy consumption and cannot remove lignin. A study [24] compared the effects of four mechanical comminution methods, including ball mills, on the energy production of biomass enzymolysis, respectively, and centrifugal milling was the most efficient from the point of view of energy efficiency. In treating thin-walled cells (PC) and vascular bundle cells (VB) from bamboo wastes by microwave liquefaction, the final amount of cellulose-rich cells obtained was 34.9 ± 1.0% and 43.8 ± 1.2%, respectively [25]. Biological pretreatment of biomass raw materials is considered environmentally friendly and not costly, but the treatment time is long. Someone [26] treated Japanese red pine with three white rot fungi, and after eight weeks of treatment with the most efficient S. hirsutum, the sample lost 10.7% of its mass. Compared to physical and biological methods, the chemical method is the most used method due to its low cost, speed, and efficiency. Researchers [27] investigated the effect of hydrothermal treatment temperature on moso bamboo pellets (BP) and PVC/BP composite and proved that the hydrothermal method has a significant effect on the removal of hemicellulose and pectin, and the content of cellulose reaches 67.91% at a hydrothermal temperature of 280 °C. Zhu et al. [28] investigated the effects of alkali and acidified sodium chlorite on polymers in moso bamboo in terms of tissue and cellular extent with the help of FTIR and Raman spectroscopy, respectively. The results proved that alkali treatment showed high sensitivity to non-cellulosic fractions, such as lignin, within the intercellular layer. Acidified sodium chlorite mainly acted on the lignin fractions of the secondary wall. However, combining alkaline and sodium chlorite was considered the most effective method for removing non-cellulosic substances [29]. In the alkaline environment created by NaOH and KOH, cleavage of ester and ether bonds occurs, resulting in the removal of hemicellulose and lignin, and then in the acidic environment produced by acetic acid, sodium chlorite produces CIO_2_, which results in the further removal of non-cellulosic fractions [30]. The removal of hemicellulose by alkali treatment can reach 72%, and the removal of lignin by chlorite is at 96% in the treatment of flax fiber using this method [29]. However, there are no studies on the combined treatment of alkaline-acid sodium chlorite for the pretreatment of moso bamboo powder, and some of the changes that occur in this process are still unknown.

Therefore, this paper focuses on the separation of the bamboo cellulose fibers (BC) by alkaline-acidic sodium chlorite treatment, reveals the changes in the micromorphology and chemical structure of moso bamboo during this process, prepares bamboo-based all-cellulose composite films by dissolving the BC in NaOH/urea aqueous solution, and explores the possibility of replacing the plastic packaging film with this biomass film.

## 2. Materials and Methods

### 2.1. Materials

Moso bamboo powder (60–80 mesh) from Suichang Hanya E-commerce Co. (Lishui, China). Sodium hydroxide, sodium chlorite, acetic acid, epichlorohydrin (ECH), and urea are supplied by Shanghai Aladdin Biochemical Technology Co. (Shanghai, China).

### 2.2. Pretreatment Separation of Bamboo Cellulose Fibers

**Figure 1:**
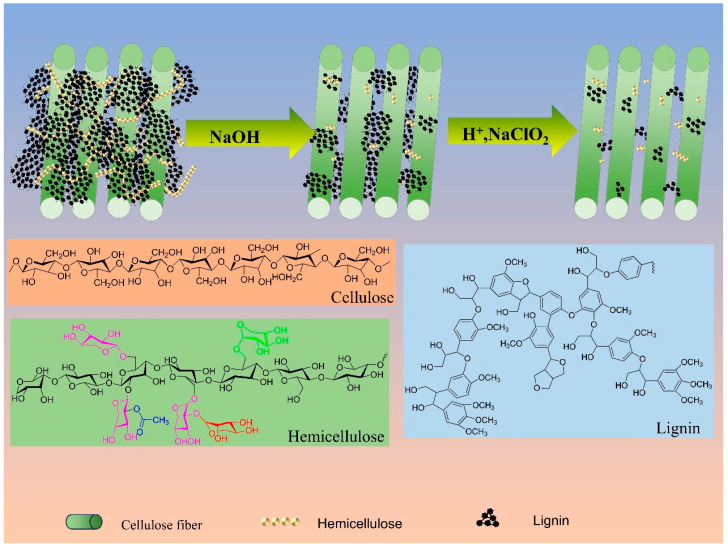
Schematic diagram of the role of pretreatment.

The purchased bamboo powder was sieved through a 60-mesh sieve, dried thoroughly in an oven at 60 °C, and then stored as BP. The BP was treated with 6 wt% NaOH at a ratio of 1:30, treated twice at 100 °C, washed several times with purified water, dried in an oven at 60 °C, and recorded as BN. The BN was treated with 1.5 wt% NaClO_2_ at 75 °C, and the pH of the solution was adjusted to about 4–5 with ethanoic acid until the fibers turned white, then washed several times with purified water until the filtrate became neutral. Finally, the samples were dried in an oven and stored in a dry tray for reserve, and the bleached sample was recorded as BC. This treatment was slightly adapted from the previous work of Thipchai [31]. The separation mechanism is shown in Schematic Figure 1.

### 2.3. Preparation of Composite Films

Three thin films with different BC contents were prepared, labeled as BC_2_, BC_3_, and BC_4_, respectively. Using BC_4_ as an example, NaOH, urea, and water were mixed at a weight ratio of 7:12:81, pre-cooled to −12.5 °C as a solvent for cellulose, then 4 g of BC sample was quickly added, stirred at 16,000 rpm for 5 min, then 3.5 mL of ECH was added. The treatment was continued under the same conditions for 5 min to obtain a cellulose solution. The solution was then degassed by centrifugation at 6000 rpm for 10 min; the supernatant was poured into a mold, coagulated, placed in a pure water bath, changed the water several times, and dried at room temperature to obtain BC_4_. BC_3_ and BC_2_ were prepared using the same procedure as BC_4_.

### 2.4. Characterizations

The sample morphology was observed by a field emission scanning electron microscope (SU-9000, Hitachi, Kyoto, Japan). Fourier transform infrared (FT-IR) spectra were obtained by an infrared spectrometer (Nicolet 380, Thermo, Waltham, MA, USA) in ATR mode. XRD analysis was performed with an X-ray diffractometer (D8 Advance, Bruker, Karlsruhe, Germany). The thickness of the films was obtained by a thickness gauge (CHY-HS, Sumspring, Jinan, China). The surface morphology was observed using an optical microscope (N-10E, Jiang Nan, Nanjing, China). The fiber diameter was counted with the help of a fiber fineness analyzer (YG02C, Changzhou Xin Fang Instrument, Changzhou, China). Roughness was analyzed with the help of a step meter (Dektak XT, Bruker, Karlsruhe, Germany). The mechanical properties of the films were analyzed on a tensile testing machine (HYTECH-03, Shanghai Qingyiyuan Technology Co., Ltd., Shanghai, China), where the samples were first cut into rectangular strips of 10 mm width and 50 mm length and tested according to the ASTM standard D638, with the span selected to be 20 mm and the loading speed selected to be 1 mm/min, and each sample was tested more than three times [32]. Optical transmittance tests were carried out on three all-cellulose composite films with different BC contents in the 400–800 nm range using a UV-Vis spectrophotometer (UH-4150, Hitachi, Kyoto, Japan) with a 20 × 20 mm sample size. The hydrophilicity of the film was determined using a contact angle analyzer (OCA50, Dataphysics-TP50, Stuttgart, Germany) by cutting the sample into 10 × 10 mm squares, affixing it to the surface of the glass plate with a water droplet volume of 3 μL, and reading contact angle data for 0, 60, 90, 120, and 180 s. Thermal properties were tested using a combined TG/DTA thermogravimetric thermal analyzer (labsys evo, SETARAM, Lyon, France), which was used to increase the temperature from room temperature to 600 °C at a ramp rate of 10 °C/min in a nitrogen atmosphere. The biodegradation experiment was based on previous research methods [33]. The venue is the campus garden of Beijing University of Technology. The cut cellulose film (4 × 8 cm) was sandwiched between two pieces of nylon cloth to facilitate sample removal and then buried in a soil layer about 10 cm deep for removing the sample on day 7 and day 14, respectively. Under the same treatment conditions, the polyethylene film was selected as the control group, and the surface morphology of the film was observed by optical and scanning electron microscopy after washing.

## 3. Results and Discussion

### 3.1. Preparation of Bamboo Cellulose Fibers and Composite Films

**Figure 2:**
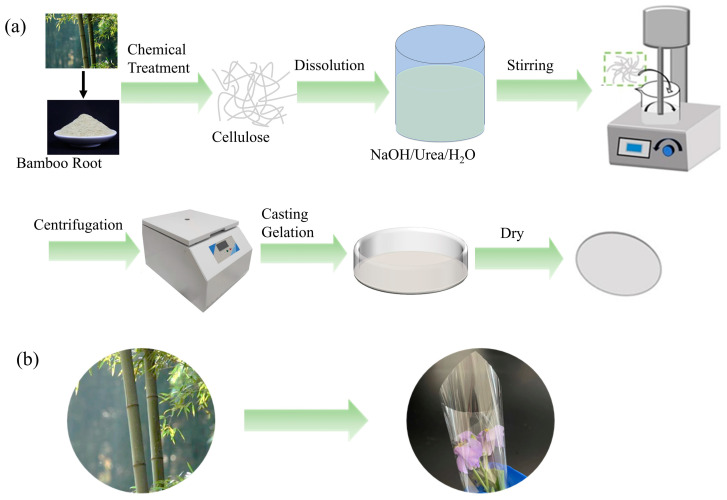
(**a**) Flow chart of the preparation of composite films and (**b**) schematic diagram of film packaging effect.

Alkaline-acidic sodium chlorite was used to pretreat moso bamboo powder to extract the BC and prepare composite films, and Figure 2a shows the experimental flow chart. Firstly, the moso bamboo powder was treated with NaOH to separate the connecting bonds between hemicellulose and lignin, to dissolve the hemicellulose component in the biomass material, and to promote the separation of hemicellulose from the raw material in the form of glycan [34]. During this time, lignin was also involved in the reaction, in which the breaking of the carbon-carbon (C-C) and ether bonds (C-O-C) of the phenylpropane unit produced small molecules and soluble lignin fragments, which were the basis for lignin removal [35]. However, the lignin structure contained many C-C bonds and aromatic groups, and alkali treatment has a limited disruptive effect [36]. For lignin removal, there are three mainstream methods, including sodium chlorite, sodium sulfite/sodium hydroxide, and acetylation for lignin retention, of which the most commonly used and fastest treatment is the acidic sodium chlorite method. Sodium chlorite was heated and decomposed under acidic conditions to produce substances such as Cl_2_, ClO_2_, and water, among which ClO_2_ had a strong destructive effect on the -C-C and -C-O-C bonds connecting the lignin molecules, thus removing the lignin components in biomass feedstocks. Previous studies have shown that the content of cellulose components in untreated moso bamboo was about 40–65%, and the cellulose content was significantly enhanced after pretreatment, as shown in Table S1. The cellulose content at different treatment stages was measured using the nitrate ethanol method by treating the bamboo powder with alkaline-acidic sodium chlorite [37], and the results of the measurements are presented in Figure S1.

The low-temperature system is critical in preparing all-cellulose composite films using NaOH/urea aqueous solution. Large clusters of solvent molecules at low temperatures break cellulose molecules’ intramolecular and intermolecular hydrogen bonds, thereby dissolving cellulose [38,39]. Generally, the raw material of dissolved cellulose needs to undergo time-consuming and laborious chemical or mechanical treatments after the bleaching process to obtain a cellulose state with higher purity and smaller particle size [40,41,42]. As shown in Figure 2b, the BC was the raw material, the dissolved portion formed the matrix, and the undissolved portion became the reinforcing phase; this cellulose reinforcing phase and cellulose matrix were compatible, resulting in the all-cellulose composite films with excellent mechanical properties and transparency.

### 3.2. Morphology and Structure of Cellulose Fibers and Composite Films

**Figure 3:**
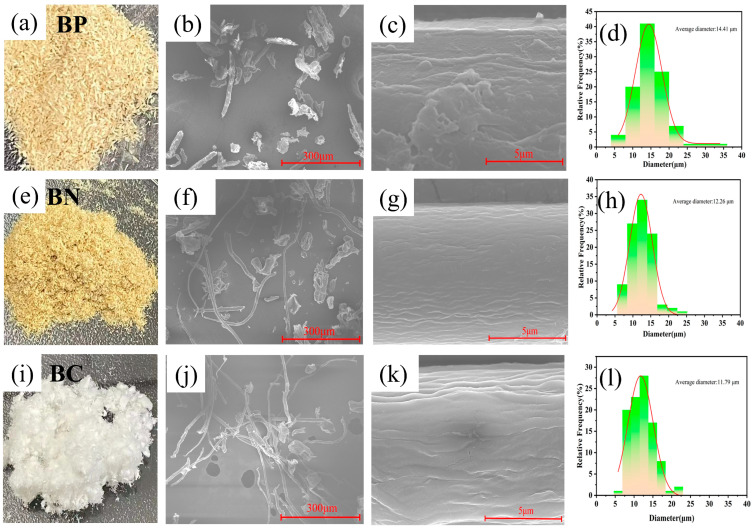
Changes in the morphology and size of the samples during the pretreatment phase. (**a**–**d**) BP; (**a**): optical image, (**b**,**c**): SEM images, (**d**): diameter statistic, average diameter 14.41 µm; (**e**–**h**) BN; (**e**): optical image, (**f**,**g**): SEM images, (**h**): diameter statistic, average diameter 12.26 µm; (**i**–**l**) BC; (**i**): optical image, (**j**,**k**): SEM images, and (**l**): diameter statistic, average diameter 11.79 µm.

Figure 3 shows the changes in the morphology of biomass raw materials during the pretreatment. The untreated bamboo powder particles BP exhibited a light yellow color (Figure 3a) after BP was treated with an alkaline solution, which removed hemicellulose and part of lignin; the color of BN was more yellow than that of BP (Figure 3e). Then, after bleaching treatment, the BC became white (Figure 3i). In the SEM image (Figure 3b,c), the BP existed in the form of thin-walled cells and bamboo fibers [43]. When viewed in magnification, the BP showed inhomogeneity with an average diameter of 14.41 µm (Figure 3d) due to substances such as pectin, lignin, and hemicellulose. After alkali treatment (Figure 3f,g), the fiber diameter decreased dramatically to 12.26 µm (Figure 3h, with a relatively smooth and uniform fiber surface. In addition, it has been pointed out that under alkali treatment conditions, the cell wall loses its matrix, and cellulose microfibrils are prone to aggregation, which produces twisting and shrinkage effects to some extent [44]. In the BC obtained from the final bleaching treatment, the fiber diameter was further reduced to 11.79 µm (Figure 3l), the wrinkles on the surface of single fibers became more and more apparent, and the microfilaments were aggregated under the action of hydrogen bonding to form larger microfibrillar units [45].

**Figure 4:**
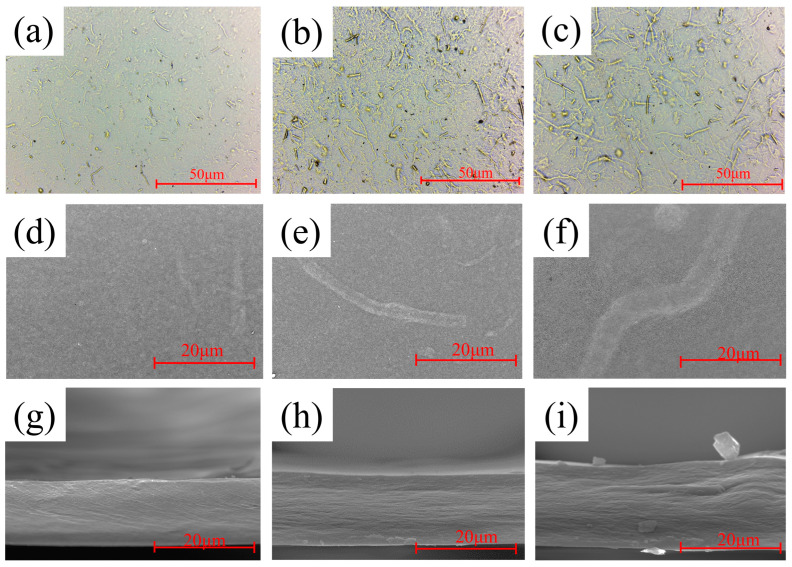
Microscopic morphologies of BC_2_, BC_3_, and BC_4_: (**a**–**c**) surface morphology of BC_2_, BC_3_ and BC_4_ under the optical microscope; (**d**–**f**) surface morphology of BC_2_, BC_3_, and BC_4_ under electron microscope; and (**g**–**i**) cross-sectional morphology of BC_2_, BC_3_, and BC_4_ under electron microscope.

The morphology of all-cellulose composite films with different contents is shown in Figure 4, with BC_2_ containing the least undissolved fractions and BC_4_ the most under both light and electron microscopy; BC_2_ has a smoother surface and a denser cross-section. As the BC content increases, there is a significant increase in undissolved and incompletely dissolved fibers in the film, which can be detected on the surface and sides of the film. The fibers observed in BC_4_ are significantly larger in diameter and length than those in BC_3_, as shown in Figure 4d–i. The undissolved reinforcement phase and the dissolved matrix are tightly bound, and no significant porosity is observed around them, indicating good compatibility between them. In Table S2, the thickness data of the films are supplemented.

**Figure 5:**
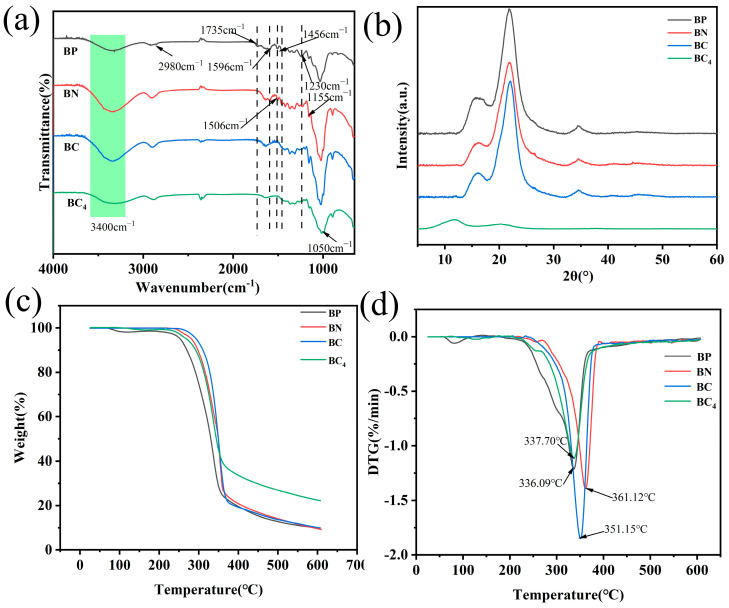
FTIR (**a**), XRD (**b**), TGA (**c**), and DTG (**d**) curves of BP, BN, BC, and BC_4_.

Figure 5a shows the FTIR spectra of BP, BN, BC, and BC_4_, which had significant similarities. The two characteristic peaks appearing at 1052 cm^−1^ and 1155 cm^−1^ were the stretching motions of the C-O structure in the glucose monomers. The peaks here did not change significantly after pretreatment, and the vibrational peaks demonstrated by the basic structural units of cellulose were still maintained. At around 3400 cm^−1^, a broad peak appeared in four samples, which was the result of the stretching vibration of -OH, while the characteristic absorption peak at 2980 cm^−1^ represented the stretching vibration of -CH_2_ in cellulose, lignin, and hemicellulose [42]. The characteristic absorption peak at 1735 cm^−1^ was evident in the untreated bamboo powder particles, which represented the C=O stretching vibration of carboxyl and acetyl groups in hemicellulose, and the characteristic absorption peak here disappeared after NaOH treatment, indicating that NaOH treatment was influential in the removal of hemicellulose [46,47]. The characteristic absorption peak at 1506 cm^−1^ was related to the stretching vibration of the benzene ring skeleton of cellulose-rich cells, indicating that lignin was present in both untreated and NaOH-treated samples, only that the peak there showed signs of weakening after NaOH treatment. The peak at this location disappeared after bleaching treatment, which proved that NaOH and acidic NaClO_2_ have significant effects on the removal of lignin [46]. The absence of the characteristic absorption peaks of lignin (1230 cm^−1^, 1456 cm^−1^, 1506 cm^−1^, 1596 cm^−1^) in the infrared spectra after bleaching treatment proved the effective removal of lignin by the pretreatment process. The overall trend of BC_4_ regenerated by dissolution was the same as before, which proved that the functional groups did not change chemically during the process, and the broad peak at 3500 cm^−1^ proved that the -OH structure was still present in the films. The shift in the characteristic peak near 1430 cm^−1^ corresponded to the cellulose type II crystal structure, consistent with the results observed in XRD [48].

The XRD patterns of the samples at the pretreatment stage and the film samples are displayed in Figure 5b. There was no apparent difference between BP, BN, and BC, while BC_4_ underwent a prominent change, indicating that the pretreatment had no significant effect on the crystal structure of the bamboo powder particles; however, dissolution regeneration destroys the original cellulose structure. The BP, BN, and BC samples showed prominent diffraction peaks around 2θ = 14.5°, 16°, 22°, and 34.5°, which were typical of cellulose type I structure, corresponding to the (1–10), (110), (200), and (004) crystal planes of monoclinic cellulose I, respectively. It was calculated that the crystallinity of BP, BN, and BC increased significantly with the pretreatment, indicating that with the pretreatment process, the chemical reaction removed the amorphous components in the bamboo powder particles and promoted the rearrangement of disordered cellulose molecules, which in turn caused the crystallinity of the samples to climb up [43,49]. The peaks observed in BC_4_ around 2θ = 12.0° and 20.5° were typical cellulose II crystalline structure peaks [50,51], the absorption peak at 12° was the result of the shift in the (1–10) crystallographic plane, and the diffraction peak at 20.5° was the overlap of the (110) and (200) diffraction planes. The disappearance of the (004) crystallographic plane suggested that the occurrence of the dissolution process drove the transformation of the cellulose I structure to the cellulose II structure. All-cellulose composite film’s lower crystallinity than the cellulose fraction that had not undergone solubilization may be attributed to the irregular and disordered rearrangement of the cellulose molecular chains during the regeneration process, making it difficult to form a highly ordered structure.

TGA and DTG analysis are performed for different pretreatment stages and BC_4_ samples_,_ as shown in Figure 5c,d. The mass loss between room temperature and 100 °C was attributed to the absorption of water molecules from the air. BP, BN, and BC decomposition started at 230 °C, 265 °C and 273 °C, respectively. The treated samples, BN and BC, possessed higher thermal stability compared to BP, which was strongly related to the removal of non-fibrous fractions, such as lignin and hemicellulose; this is similar to previous findings [49,52,53]. In the final residual mass, untreated BP was the least, and BC_4_ was the most, about 25%, which was much lower than the ideal residual carbon mass of 44.4% after the thermal decomposition of cellulose H and O. This was inextricably related to the complex side reactions induced by the thermal decomposition process in the actual situation. The higher residual mass of BC_4_ was related to the crystalline structure of cellulose II formed by solvation and regeneration, which was much closer to the ideal situation [54,55]. The temperature corresponding to the rate of weight loss at the fastest point of the cellulose film regenerated by dissolution is found to be lower than that of the pretreated completed biomass sample in Figure 5d, and this decrease in thermal stability was inextricably linked to the decrease in film crystallinity [56]. Although the degradation temperature of BC_4_ film is weaker than that of PE film (442 °C) [57], it is still at a high level compared to other polymer films [58].

### 3.3. Properties of All-Cellulose Composite Films

**Figure 6:**
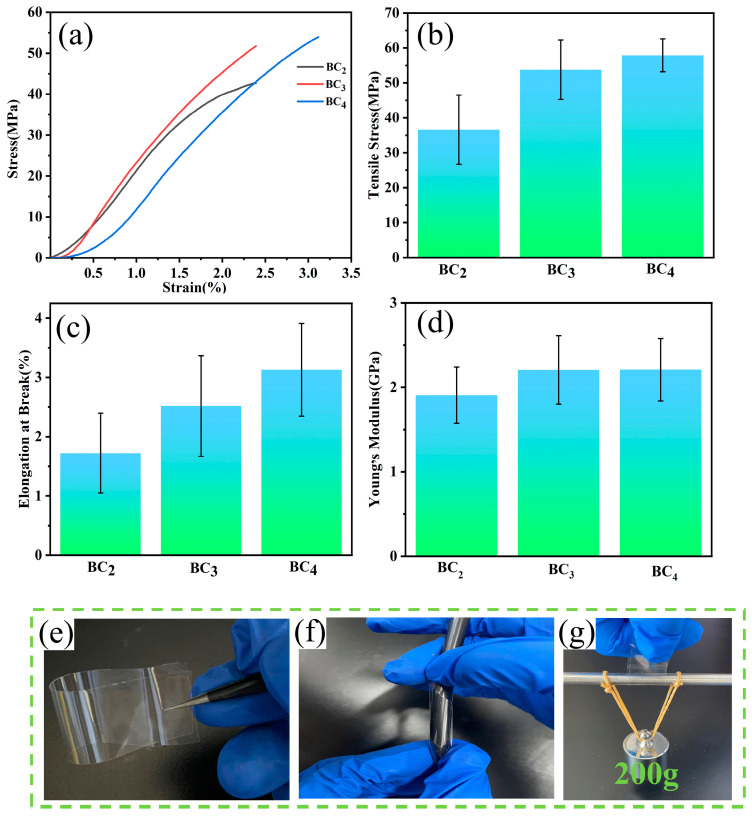
Mechanical properties of BC_2_, BC_3_, and BC_4_: (**a**) stress–strain curves; (**b**) tensile strength; (**c**) elongation at break; (**d**) Young’s modulus; digital photograph of BC_4_ (2 × 5 cm) (**e**) folding; (**f**) torsion; and (**g**) easily withstands 200 g weights.

The mechanical properties of packaging films were crucial in ensuring the integrity of the packaged product, and Figure 6 demonstrates the mechanical properties of the films with different cellulose content. The tensile strength and elongation at the break of the films are enhanced with the increase in cellulose content (Figure 6a–c). The tensile strength of BC_4_ is about 58 MPa, which is 68% higher than that of commercial PE cling film, and Young’s modulus of BC_2_, BC_3_, and BC_4_ films also far exceeds that of PE films (113.25 MPa) [59]. The increase in content caused the dissolution system to contain more cellulose molecules, which were intertwined and interdependent, resulting in higher-density films at higher content. In addition, the high content of cellulose molecules contributed to the occurrence of slip between molecules, which led to the increase in the elongation at break, and the incompletely dissolved cellulose component played the reinforcing body role, further enhancing the mechanical properties. As a result, highly concentrated cellulose films possessed higher tensile strength and elongation at break [60]. However, some studies have shown that this enhancement of mechanical properties with increasing content was not without limits. Previous studies have found that when the content exceeded 5% [55], the high content led to an increase in the viscosity of the solution, deterioration of the fluidity, and insufficient homogeneity of the casting, which led to an increase in the internal defects of the film, and the hydrogen bonding between cellulose molecules cannot be opened at a high concentration, which made it challenging to play a role, and the excessive cellulose chains between the entanglement limited the solubility of cellulose and affected the molecular orientation, leading to a decrease in the mechanical properties [61]. The flexibility and tensile properties of the films are demonstrated in Figure 6e–g, respectively, which can be flexibly folded, twisted, and easily withstand the weight of 200 g.

**Figure 7:**
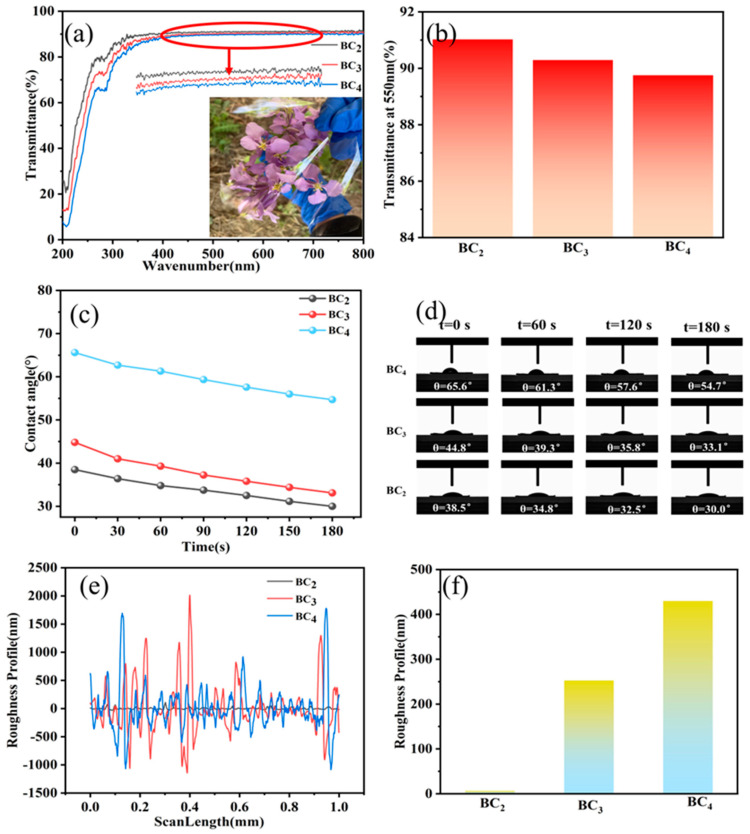
Transparency, hydrophobic properties, and roughness profiles of BC_2_, BC_3_, and BC_4_: (**a**) transmittance curves, inset area magnified image at 400–600 nm and an optical photograph demonstrating the transparency of the film; (**b**) transmittance values at 550 nm; (**c**) contact angle curves; (**d**) water contact angle images of droplets on the surface of the composite films with time; (**e**) roughness curves; and (**f**) films surface roughness values.

The transparency of packaging materials is crucial, and high transparency can display packaged products well and significantly increase their commercial value. Figure 7a demonstrates the transparency of films with different content, from which it can be seen that the higher the content, the lower the transparency of the films, but all of them showed high levels. The transmittance of BC_4_, BC_3_, and BC_2_ at 550 nm was 89.75%, 90.29%, and 91.03%, respectively, which was higher than that of the films prepared using disposable paper cups in the solidification bath of water and ethanol [19]. It still offers significant advantages over PE films prepared by stretching processes from mLLDPE and LDPE [62]. The decrease in transparency due to the increase in content may be attributed to the high content being accompanied by an increase in film thickness and porosity, which led to enhanced scattering of light in the film and a decrease in transparency. However, the scattering of light by the large undissolved and incompletely dissolved cellulose fractions presented by BC_4_ under optical microscopy was further enhanced but still showed high transparency in the flower packs.

To better reflect the hydrophilic/hydrophobic properties of the films, a contact angle (CA) analysis was carried out. From Figure 7c,d, the BC_4_ has the largest contact angle, which reached 65.6° when it is just in contact with a water droplet. The hydrophobicity of the material corresponds to the large contact angle. Therefore, the BC_4_ had superior hydrophobicity. Even after contacting the water droplets for 3 min, it still maintained a high contact angle of 54.7°, which was slightly lower than that of the commercially available general PE cling film (71.6°) but was still higher than that of the other polymer packaging materials [59].

The film’s roughness is shown in Figure 7e,f. From Figure 7e, it can be seen that BC_4_ moves up and down along the peaks and valleys most vigorously in the range of 1 mm, followed by BC_3_, and the smallest one is BC_2_, which indicates that the roughness of BC_4_ is the largest and the average roughness of BC_2_ is the smallest; however, the roughness of BC_4_ is much lower than that of the surface of the CNF film (300 μm) [63], which may be due to the presence of many incompletely dissolved components in BC_4_, which are randomly arranged and distributed, showing an undulating structure in the 2 μm range. This rough structure is the main reason for the hydrophobicity of the films.

**Figure 8:**
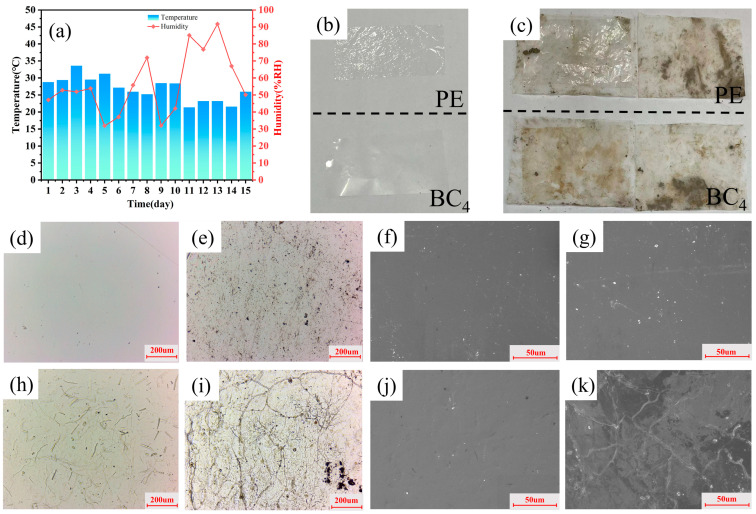
Degradation properties of PE and BC: (**a**) Temperature and humidity conditions in the experimental locality; the histogram corresponds to the temperature scale on the left axis, and the line chart with dots corresponds to the humidity curve on the right axis; (**b**,**c**) macroscopic morphology of PE and BC_4_ films before and after degradation (up: PE film; down: BC film); (**d**–**g**) Optical microscope images (**d**,**e**) and SEM images (**f**,**g**) of PE film before and after degradation; (**h**–**k**) Optical microscope images (**h**,**i**) and SEM images (**j**,**k**) of BC_4_ film before and after degradation.

Figure 8 illustrates the degradation behavior of BC_4_ film and control PE film. Figure 8a shows the local temperature and humidity during the test, and Figure 8b,c shows the macroscopic topography of the two different material films before and after the experiment. Before the experiment, the PE and BC_4_ films were morphologically intact and smooth. After 7 and 14 days of degradation testing, the PE film remains intact and can be easily removed from the covered nylon cloth, while the BC_4_ film adheres firmly to the nylon cloth. After 7 days, it could not be completely removed from the nylon cloth. After 14 days, the tiny lumps could not be removed, which made it impossible to observe the degradation ability of the film by the weight loss of the sample. However, in optical photographs and SEM images, it was observed that on day 7 of the degradation test, a large number of fungal-like substances appeared on the surface of BC_4_, similar in morphology to the fungi of biodegradable cellulose materials that had been isolated, purified, and identified in previous studies [64]. Therefore, it is certain that the presence of these fungi causes the degradation of the BC membrane.

As shown in Table 1, the films prepared in this study are not inferior to those prepared using cellulose nanocrystals and smaller nanoscale cellulose nanofibers regarding mechanical properties, transparency, and hydrophobicity. These excellent properties make the films prepared in this study promising for food, electronics, biomedical, and other packaging material applications.

## 4. Conclusions

This study systematically investigated the process of extracting bamboo cellulose fibers by treating moso bamboo powder with a combination of alkali and acidic sodium chlorate, and the possibility of using bamboo cellulose fiber as a raw material for the preparation of transparent and degradable films with excellent mechanical properties to replace PE films was discussed. During the pretreatment process, substances such as lignin and hemicellulose were effectively removed, and the final BC content obtained was as high as 74.02%. Whole cellulose composite films were prepared by adding the obtained BC to an aqueous NaOH/urea solution at low temperatures. The mechanical properties of the films were significantly improved with the increase in the added amount; the hydrophobicity was also improved; and the transmittance in the visible range was reduced but remained at a high level. In addition, in the degradation experiment, many fungi appeared on the surface of the BC film on the seventh day compared with the PE film, which indirectly proved that the BC film had good degradation properties. In summary, the extraction of bamboo cellulose fiber as a raw material for the preparation of all-cellulose composite film by simple and efficient alkali and acidic sodium chlorite pretreatment of moso bamboo powder may be expected to be applied as an environmentally friendly material in the field of packaging, which provides new possibilities for green innovation and development of the packaging industry.

## Figures and Tables

**Table 1:** Comparison of the properties of packaging films prepared in this study with other films reported in the literature.

Sample	Method	Mechanical Property			Transmittance (%)			Contact Angle (°)	Ref.
		Tensile Strength (GPa)	Elongation at Break (%)	Elastic Modulus (GPa)	550 (nm)	660 (nm)	750 (nm)		
CMC/CNF10%	Blend	49.2	26.6	1.33	\	86.2	\	23.9	[15]
CMC/CNF3%		46	28.4	1.27	\	86.7	\	27.1	
CMC	Blend	36.29	\	0.23	\	\	\	61.2	[59]
Ag—2MI @ CMC		61.12	\	0.34	\	\	\	68	
Dmm	Regeneration	69.20 ± 0.05	7.14 ± 0.03	0.6 ± 0.02	\	\	58.2	36	[65]
Dsf		81.09	10.98	0.36	\	\	68	48	
Cellulose film	Regeneration	32.8	4	\	Transparent			\	[33]
RC6	Regeneration	77.22	14	\	25.8	\	\	57.2	[66]
RC0		54.69	5.9	\	89.5	\	\	36.2	
CNF20%	Blend	10.98	2.78	0.51	\	\	\	63.16	[67]
CNF30%		12.68	2.77	0.56	\	\	\	61.81	
CNF50%		11.51	2.13	0.59	\	\	\	68.63	
BC_4_	Regeneration	57.9	3.1	2.2	89.86	89.97	89.68	65.6	This Work

## Data Availability

Data are contained within the article.

## Supplementary Materials

The following supporting information can be downloaded at: https://www.mdpi.com/article/10.3390/polym16233361/s1, Figure S1: Cellulose content at different pretreatment stages; Table S1: Chemical composition of the components of moso bamboo before and after treatment; Table S2: Film thickness data for different concentrations. References [68,69,70] are cited in the supplementary materials.
