# Supplementary material for: Alkaline–Acidic Sodium Chlorite Pretreatment of Bamboo Powder for Preparation of Excellent Mechanical, Transparent, and Biodegradable Films
Source: Polymers (Basel). 2024 Nov 29;16(23):3361. doi: 10.3390/polym16233361 (PMC11644612; doi:10.3390/polym16233361)
Supplement: Supplementary file 1 [file polymers-16-03361-s001.zip › polymers-3326609-supplementary.pdf]

Alkaline–Acidic Sodium Chlorite Pretreatment of Bamboo Powder for Preparation of Excellent Mechanical, Transparent, and Biodegradable Films

Jing Wang [a], Ling Sun[a, b*], Yingying Chu [a], Yongqi Ou [a], Bowen Liang[a], Zijian Shan [a], Changbao Han [a], Hui Yan[a]

a. Key Laboratory of Advanced Functional Materials, Ministry of Education, Faculty of Materials and Manufacturing, Beijing University of Technology, Beijing 100124, China

b. Beijing Guyue New Materials Research Institute, Beijing University of Technology, Beijing 100124, China

**Corresponding author.E-mail:: sunling@bjut.edu.cn (L. Sun)*

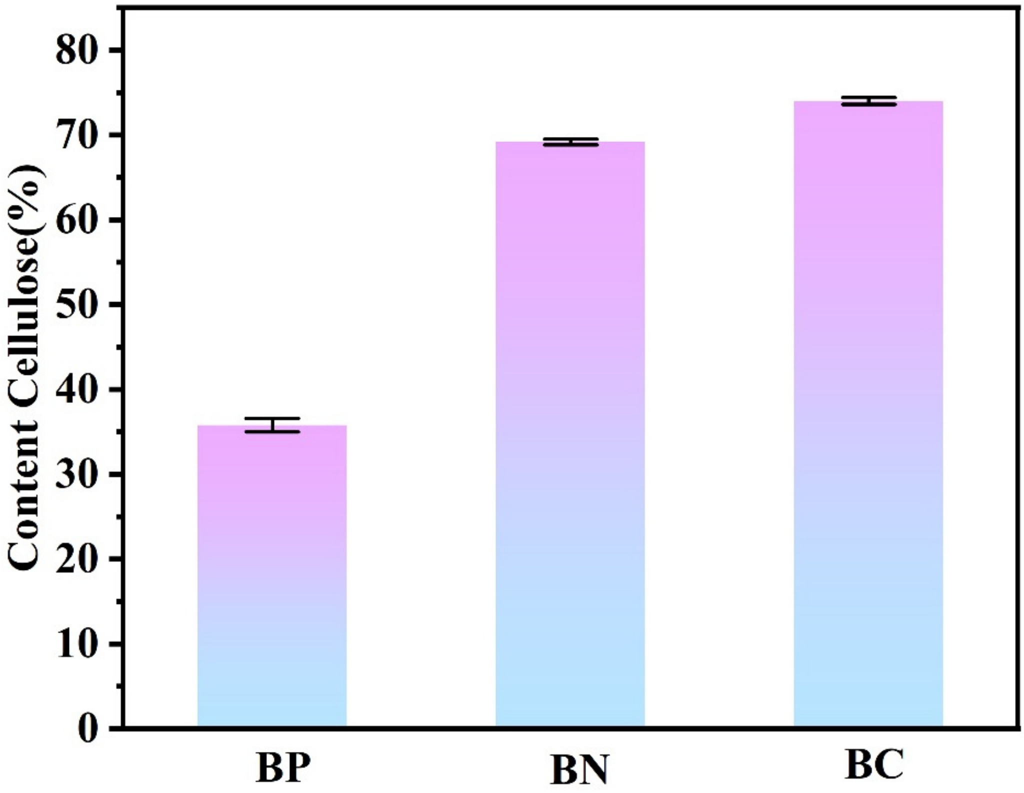

Figure S1. Cellulose content at different pretreatment stages

Table S1 Chemical composition of the components of moso bamboo before and after treatment

Sample	Processing method	Cellulose(%)	Hemicellulose(%)	Lignin(%)	References
Original bamboo	1. Microwave liquefaction 2. Bleaching 3. Alkali treatment	41.72±2.37	22.86±2.19	20.91±0.24	[1]
Treated bamboo		83.67±2.69	13.97±1.67	0.13±0.06	
Bamboo shavings	1. Soxhlet-extracted 2.NaOH treatment 3.H_2O_2 treatment	45.32	18.67	25.82	[2]
Treated bamboo		73.64	14.11	3.23	
Bamboo chip	1. glycerol treatment 2. Ascrew extruded	51.7	21.2	25.3	[3]
Treated bamboo		59.6	19..5	21.3	
Bamboo processing residues	1. Soxhlet-extracted 2. Bleaching 3. Alkali treatment	68.76	28..45	1.01	[4]
Bamboo pulping fibers		74.36	25.29	0.08	

Table S2 Film thickness data for different concentrations

Sample	BC_2	BC_3	BC_4
Thickness（μm）	20.03±0.97	24.36±1.52	36.3±1.63

Reference

1. Xie, J.; Hse, C.-Y.; De Hoop, C.F.; Hu, T.; Qi, J.; Shupe, T.F. Isolation and characterization of cellulose nanofibers from bamboo using microwave liquefaction combined with chemical treatment and ultrasonication. *Carbohydr. Polym.* **2016**, *151*, 725-734.
2. Ding, X.; Dai, R.; Chen, H.; Shan, Z. Gelatin as green adhesive for the preparation of a multifunctional biobased cryogel derived from bamboo industrial waste. *Carbohydr. Polym.* **2021**, *255*, 117340.
3. Lu, H.; Zhang, L.; Liu, C.; He, Z.; Zhou, X.; Ni, Y. A novel method to prepare lignocellulose nanofibrils directly from bamboo chips. *Cellulose* **2018**, *25*, 7043-7051.
4. Wang, H.; Zhang, X.; Jiang, Z.; Li, W.; Yu, Y. A comparison study on the preparation of nanocellulose fibrils from fibers and parenchymal cells in bamboo (Phyllostachys pubescens). *Ind. Crops Prod.* **2015**, *71*, 80-88.
